# Liquid Phase and Microwave-Assisted Extractions for Multicomponent Phenolic Pattern Determination of Five Romanian *Galium* Species Coupled with Bioassays

**DOI:** 10.3390/molecules24071226

**Published:** 2019-03-28

**Authors:** Andrei Mocan, Alina Diuzheva, Sabin Bădărău, Cadmiel Moldovan, Vasil Andruch, Simone Carradori, Cristina Campestre, Angela Tartaglia, Marta De Simone, Dan Vodnar, Matteo Tiecco, Raimondo Germani, Gianina Crișan, Marcello Locatelli

**Affiliations:** 1 Department of Pharmaceutical Botany, “Iuliu Haţieganu” University of Medicine and Pharmacy, 400012 Cluj-Napoca, Romania; mocan.andrei@umfcluj.ro (A.M.); moldovan.cadmiel@yahoo.com (C.M.); gcrisan@umfcluj.ro (G.C.); 2 Department of Analytical Chemistry, Pavol Jozef Šafárik University, SK-04180 Košice, Slovakia; adyuzheva@gmail.com (A.D.); vasil.andruch@gmail.com (V.A.); 3 Department of Environmental Sciences, Babeș-Bolyai University, 400084 Cluj-Napoca, Romania; alexandru@transsilvanica.net; 4 Department of Pharmacy, University “G. D’Annunzio” of Chieti-Pescara, 66100 Chieti, Italy; simone.carradori@unich.it (S.C.); cristina.campestre@unich.it (C.C.); angela.tartaglia@unich.it (A.T.); marta.desimone@studenti.unich.it (M.D.S.); 5 Department of Food Science, University of Agricultural Sciences and Veterinary Medicine, 400372 Cluj-Napoca, Romania; dan.vodnar@usamvcluj.ro; 6 Department of Chemistry, Biology and Biotechnology, University of Perugia, 06132 Perugia, Italy; matteotiecco@gmail.com (M.T.); raimondo.germani@unipg.it (R.G.)

**Keywords:** dispersive liquid-liquid microextraction, microwave-assisted extraction, natural deep eutectic solvent, β-cyclodextrin, *Galium* species, tyrosinase inhibition

## Abstract

**Background**: Galium is a plant rich in iridoid glycosides, flavonoids, anthraquinones, and small amounts of essential oils and vitamin C. Recent works showed the antibacterial, antifungal, antiparasitic, and antioxidant activity of this plant genus. **Methods**: For the determination of the multicomponent phenolic pattern, liquid phase microextraction procedures were applied, combined with HPLC-PDA instrument configuration in five Galium species aerial parts (G. verum, G. album, G. rivale, G. pseudoaristatum, and G. purpureum). Dispersive Liquid–Liquid MicroExtraction (DLLME) with NaCl and NAtural Deep Eutectic Solvent (NADES) medium and Ultrasound-Assisted (UA)-DLLME with β-cyclodextrin medium were optimized. **Results**: The optimal DLLME conditions were found to be: 10 mg of the sample, 10% NaCl, 15% NADES or 1% β-cyclodextrin as extraction solvent—400 μL of ethyl acetate as dispersive solvent—300 μL of ethanol, vortex time—30 s, extraction time—1 min, centrifugation at 12000× g for 5 min. **Conclusions**: These results were compared with microwave-assisted extraction procedures. *G. purpureum* and *G. verum* extracts showed the highest total phenolic and flavonoid content, respectively. The most potent extract in terms of antioxidant capacity was obtained from *G. purpureum*, whereas the extract obtained from *G. album* exhibited the strongest inhibitory effect against tyrosinase.

## 1. Introduction

The use of plants for the treatment of human diseases is a centuries-old tradition, based on phytotherapy research as well as on ethnopharmacological knowledge. Recently, the use of herbal medicines applied for the prevention and/or preservation of health covers a central role in modern medicine related to the fact that these plant-derived materials avoid the classical side effects of synthetic drugs. Additionally, there are benefits of their long-term historic use—safety, accessibility, and efficacy with a wide range of therapeutic actions [1]. *Galium* is a well-known genus with many medicinal representatives that are rich sources of iridoid glycosides [2,3,4], flavonoids [5], anthraquinones [6], and small amounts of essential oils and vitamin C [7]. Recent studies showed the antibacterial, antifungal, antiparasitic, and antioxidant activities of representatives of this plant genus [7,8].

*G. verum* L., also known as Lady’s Bedstraw, is an herbaceous perennial plant, native to Europe and Asia, and used commonly in many countries’ folk medicine for a large variety of treatments. The dried plants’ aerial parts were used to stuff mattresses, and the flowers were also used to coagulate milk for cheese production [9]. The cut and dried aerial parts of the plant, ‘*Herba gallii verii*’, are used for homeopathic purposes. These are still used for exogenous treatment of psoriasis or as a tea with diuretic effect for the cure of pyelitis or cystitis [10]. Moreover, *G. verum* L. has been used as a diuretic for bladder and kidney irritation, externally for poorly healing wounds, as well as for epilepsy and hysteria in Montenegro’s traditional medicine [11]. Regarding Turkish folk medicine, it has been used for its diuretic, choleretic, antidiarrheal, and sedative effects [4]. In Romania, the plant is used in traditional medicine mainly for its diuretic, depurative, laxative, sedative, and antirheumatic effects. Additionally, in the Romanian traditional medicine, several *Galium* species are used as components of different cosmetic formulations [12]. *G. album* Mill., the “white bedstraw” or “hedge bedstraw”, is an herbaceous annual plant, cited in traditional Albanian pharmacopoeias and folk medicine for healing wounds and gingival inflammations [13]. *G. rivale*, *G. pseudoaristatum*, and *G. purpureum* (syn. *Asperula purpurea*) are less-known species, and to the extent of our knowledge, they have not been investigated yet in terms of chemical composition and antioxidant capacity, nor in terms of enzyme inhibitory potential.

Generally, the use of different extraction procedures on plant-derived material yields different biological activities. In this field, the availability of an efficient, fast, exhaustive, and reproducible extraction procedure allows obtaining a standardized starting material for food additives, nutraceuticals, and phytoformulations. For the extraction of bioactive compounds from *Galium* maceration in methanol [7] or ethanol [14], percolation in methanol [8], and ultrasound-assisted extraction [12] were applied, wherein the extraction time was varied from 30 min to one week. In order to reduce the extraction time and retain or increase the extraction efficiency, new extraction methods are required.

Liquid phase microextraction techniques are positioned as ‘green’ chemistry methodologies, which require small amounts of organic solvents. In order to make the procedures more environmentally-friendly, ionic liquids (ILs) or natural deep eutectic solvents (NADESs) are frequently used. Comparing ILs with NADESs, more advantages are on the side of NADESs due to their natural original (the main components can be sugars and organic acids), which may vary depending on analysis purpose, making them nontoxic, biodegradable, and incombustible. In comparison with NADESs, most ILs are toxic, have low biodegradability, and have high cost. Either IL or NADES can have high viscosity, so their extracts are limited for direct analysis using HPLC or GC systems [15,16,17,18].

Regarding biological activities, in the current work, a key enzyme was considered in order to further evaluate the extracts. Particularly, pigmentation is one of the most obvious phenotypical characteristics in the natural world. Between the pigments, melanin is one of the most widely distributed and is found in bacteria, fungi, plants, and animals. Melanins are heterogeneous polyphenol-like biopolymers with complex structure and color varying from yellow to black. The synthesis of melanin plays an important role in skin color and pigmentation. Tyrosinase, a copper-containing mono-oxygenase, is a key enzyme in melanin biosynthesis [19]. Skin disorders, such as melasma (facial pigmentation), scarce, and freckles, are related to excessive melanin biosynthesis. Thus, tyrosinase inhibitors are used to control or treat pigmentation disorders and are widely used in the cosmetic industry. In fact, some tyrosinase inhibitors, such as kojic acid and hydroquinones, are nowadays commercially produced, but they can present severe side effects, such as skin inflammation [20]. Hence, in recent years, more attention has been paid to the use of natural plant extracts as a safe and alternative source of tyrosinase inhibitors for cosmetic purposes.

In the present study, following our research on innovative microextraction procedures [21,22,23,24,25,26,27], different microextraction procedures were examined for the analysis of phenolic compounds in *G. verum* aerial parts, and then applied for the determination of the phenolic pattern of four other *Galium* species (*G. album*, *G. rivale*, *G. pseudoaristatum*, and *G. purpureum*). As an alternative, the microwave-assisted extraction (MAE) technique was used as a reference method [27,28,29]. To the best of our knowledge, it is the first time that microextraction techniques have been applied for the recovery and the establishment of phenolic compounds in *Galium* species.

## 2. Results and Discussion

### 2.1. Preliminary Examinations

**Figure 1:**
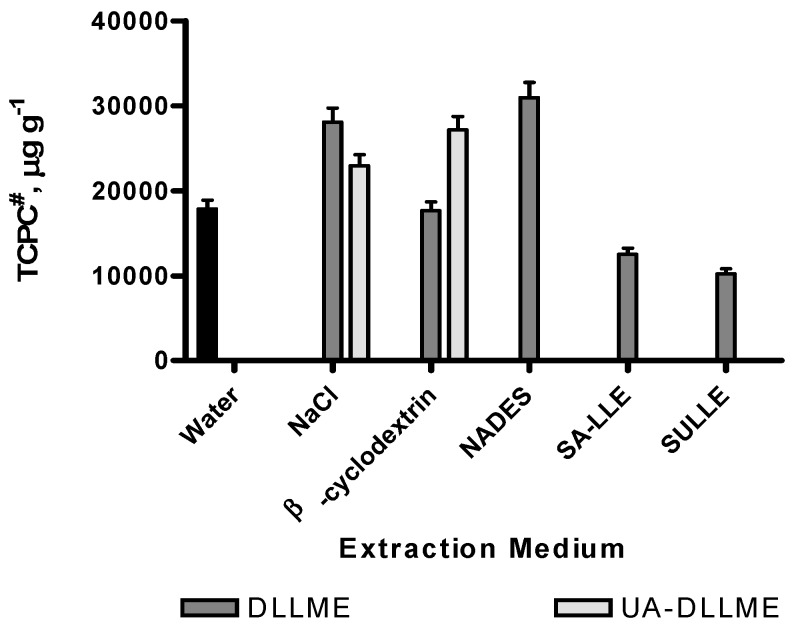
Selection of microextraction procedure. ^#^ TCPC—Total concentration of phenolic compounds. Values expressed are means ± S.D. of three measurements. All the values were statistically significant (*p* < 0.001). Raw data regarding the statistical analyses were reported in *Supplementary Materials* section S1.

Several liquid phase extraction techniques, such as DLLME, UA-DLLME in water, 10% NaCl, NADES, and 1% β-CD media, SA-LLE, and SULLE, were performed in order to select the procedure providing the better quali-quantitative multicomponent profile of phenolic compounds. The extractions were carried out as described in the experimental section. Figure 1 shows that the best results were achieved in the case of DLLME in 10% NaCl and 10% NADES media and UA-DLLME in 1% β-CD. In UA-DLLME, the phase separation was observed only with β-CD, whereas no phase separation was observed using the other additives. The notable increasing of extraction recovery using UA-DLLME with β-CD could be explained because β-CD was able to better dissolve the metabolites in the extraction solvent, contributing to an increased inclusion in its cavity of a higher amount of phenolic compounds. Therefore, DLLME in NaCl and NADES, UA-DLLME in β-CD media were selected for optimization.

### 2.2. Optimization of DLLME and UA-DLLME

Several parameters that could influence the extraction efficiency, such as solid:liquid ratio, extraction and dispersive solvent types, and volume, were selected for optimization. For UA-DLLME, ultrasonication time was also optimized.

#### 2.2.1. Optimization of Extraction Medium Concentration

**Figure 2:**
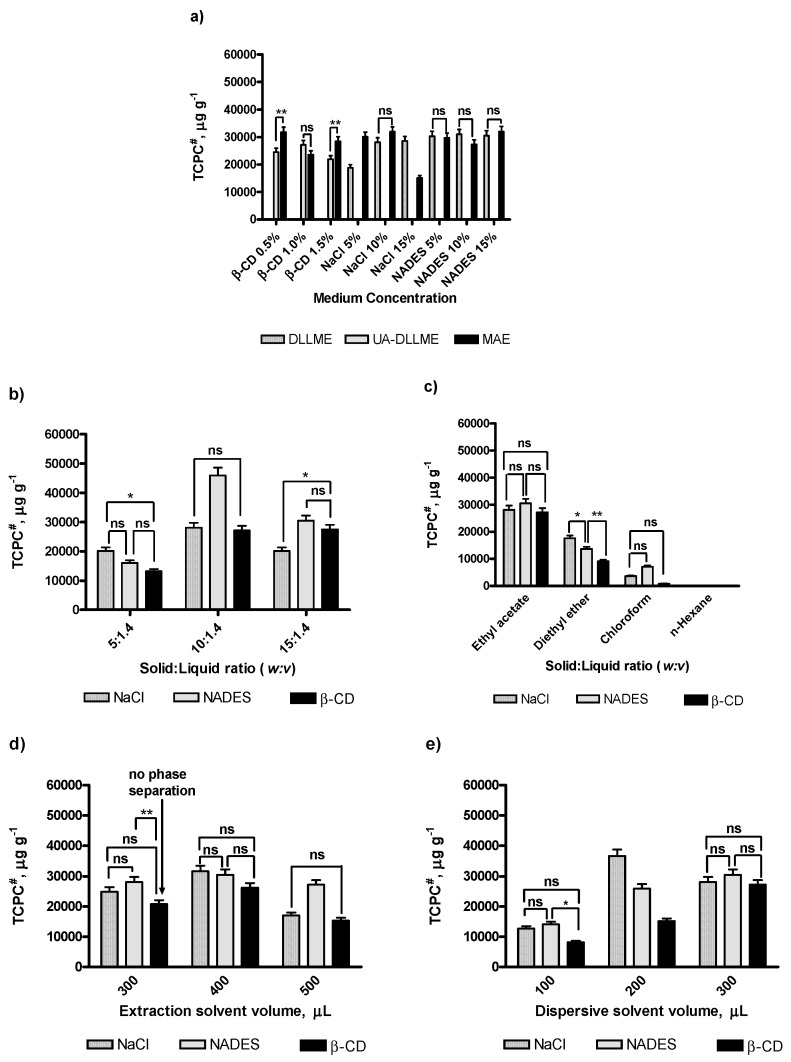
Optimization of DLLME, UA-DLLME, and MAE. (**a**) Effect of medium concentration; (**b**) Effect of solid:liquid ratio; (**c**) Selection of extraction solvent; (**d**) Effect of extraction solvent volume; (**e**) Effect of dispersive solvent volume. ^#^ TCPC—Total concentration of phenolic compounds. Values expressed are means ± S.D. of three measurements. All the values were statistically significant (*p* < 0.001), unless otherwise indicated as n.s. (not statistically significant), ** (statistically significant at *p* < 0.01), or * (statistically significant at *p* < 0.05). Raw data regarding the statistical analyses were reported in *Supplementary Materials* section S1.

The extraction medium can significantly affect the extraction yields; therefore, a series of experiments were carried out by adding 5–15% NaCl or NADES solution, or 0.5–1.5% β-CD solution into the vessel containing 10 mg of the dry herbal material. For β-CD, the concentration was lower due to their low water solubility. With 10% NaCl, 15% NADES, and 1% β-CD, the best extraction recoveries were achieved (Figure 2a). Thus, these conditions were applied in further experiments.

#### 2.2.2. Optimization of Solid:Liquid Ratio

Three solid:liquid ratios, expressed as mg/mL (5:1.4, 10:1.4, 15:1.4 *w*:*v*), were examined for their impact on the extraction efficiency. The experimental results showed that the tendency for NaCl and NADES was similar, and the maximum of the extraction recovery was reached with the ratio 10:1.4. For β-CD, with the ratios 10:1.4 and 15:1.4 (*w*:*v*), no significant differences were observed. Therefore, the optimal solid:liquid ratio was established as 10:1.4 (*w*:*v*) (Figure 2b). In fact, in the analytical chemistry workflow, if two different systems show similar data, the lower ratio is generally used because it can get the same analytical performances using a lower amount of solvents, raw material, chemicals, etc.

#### 2.2.3. Selection of Extraction Solvent Type and Volume

*n*-Hexane, ethyl acetate, chloroform, and diethyl ether were tested as potential extractants. The experiments revealed that a higher amount of phenolic compounds was extracted using ethyl acetate (Figure 2c). This could be explained by the different polarities of the extraction solvents and by the interaction with polar phenolic compounds. For instance, with *n*-hexane, a nonpolar solvent, the phenolic compounds were poorly extracted. Diethyl ether and chloroform showed similar extraction efficiency with NaCl and NADES additives, whereas the addition of β-CD did not provide an exhaustive extraction. Taking into account the high volatility of diethyl ether, it was easier to work with ethyl acetate. Therefore, ethyl acetate was selected as appropriate solvent for all samples.

To determine the optimal volume of the extraction solvent, 200, 300, 400, and 500 μL were examined. When the volume is less than 300 μL, the phase separation was not achieved in DLLME and UA-DLLME, while phase separation was reached with 300 μL or more of NaCl and NADES. In order to apply this volume amount to other solvent media, the extraction procedure was modified as follows: the extraction solvent was added in two steps, firstly 200 μL were added in order to achieve an emulsion, then an additional 100 μL of ethyl acetate were rapidly injected. The phase separation was achieved after 5 min in the rest. Applying this procedure, no phase separation in UA-DLLME in β-CD media was observed; therefore, the UA-DLLME was carried out with 400 and 500 μL of ethyl acetate. It was found that with the increase of volume of ethyl acetate, the extraction of total content of phenolics decreased. Therefore, 400 μL was selected for further study on solid samples (Figure 2d).

#### 2.2.4. Selection of Dispersive Solvent Volume

Commonly, ethanol, methanol, and acetonitrile are reported as dispersive solvents in DLLME. In this study, ethanol was selected as dispersive solvent because some food supplements, not considered in this study, of *Galium* are in ethanolic solution. Therefore, the effect of its volume (100–500 µL) on the extraction yields was tested. The results showed that the extraction efficiency was enhanced with the increase of the ethanol volume in the solution until 300 µL, while for higher volumes, phase separation was not achieved (Figure 2e).

#### 2.2.5. Optimization of Ultrasonication Time in UA-DLLME

The cyclodextrins (α, β, γ) show amphiphilic characteristics related to a hydrophilic shell and a hydrophobic cavity and could be usefully used as emulsifiers in order to enhance the extraction recovery for the target analytes. Their capacity to improve the extraction efficiency is related to their ability to reduce the interfacial tension between the two phases by an organic solvent/cyclodextrin complex located in the liquid–liquid interface. In this way, an increased contact area between the two phases was observed [30,31,32,33]. The aid of ultrasonication was generally required in order to enhance the solubility, as discussed by Saokham et al. [34] in a recent review paper. Different times have been investigated in the range of 2 to 10 min. Since 5 and 10 min showed similar responses, 5 min was selected as optimal in order to reduce the time of analysis.

### 2.3. Reference Method: Microwave-Assisted Extraction

In order to evaluate the performances of the proposed procedure, as in the comparison method, MAE was selected and carried out in the same media as LPME procedures at different concentration levels of the solvents (5–15% solution of NaCl and NADES, and 0.5–1.5% solution of β-CD). Figure 2a shows that the extraction efficiencies obtained in 10% NaCl and 15% NADES were comparable to DLLME and UA-DLLME. Therefore, the recovery of total phenolics, using LPME and MAE, was also comparable.

Following our experimental data, the optimized DLLME conditions found were: 10 mg of the sample, 10% NaCl, 15% NADES or 1% β-cyclodextrin, extraction solvent—400 μL of ethyl acetate, dispersive solvent—300 μL of ethanol, vortex time—30 s, extraction time—1 min, centrifugation at 12000× *g* for 5 min. In the case of UA-DLLME, 5 min of ultrasonication was required.

### 2.4. Total Phenolic and Flavonoid Content, Antioxidant Capacity, and Tyrosinase Inhibitory Activity

#### 2.4.1. Total Phenolic Content (TPC) by Spectrophotometric Assay

The Folin–Ciocâlteau assay was employed to determine the TPC of *Galium* extracts. The maximum TPC was registered in the ethanolic extract of *G. purpureum* (10.3 ± 0.8 mg GAE/g extract), whereas the lowest concentration was present in the ethanolic extract of *G. rivale* (1.3 ± 0.2 mg GAE/g extract). A recent study by Lakić et al. showed similar results regarding the low phenolic content of *G. verum* (2.4–5.2 mg GAE/g extract), using different extraction solvents [7].

#### 2.4.2. Total Flavonoid Content (TFC) by Spectrophotometric Assay

Results of the total flavonoid content (TFC) of the different plant materials are presented in Table 1. The highest amount for the TFC was obtained for *G. verum* extract, with a value of 8.60 ± 0.07 mg QE/g d.w., comparable with the value obtained for *G. purpureum* extract, containing 8.50 ± 0.04 mg QE/g (d.w.). According to the results of the present study, Vlase et al. reported a TPC of 5.2 ± 0.2 g/100 g for a *G. verum* extract [12] and, additionally, Lakić et al. reported values of 6.4–17.9 mg QE/g (d.w.), for *G. verum* extracts, using different solvents and extraction times [7] confirming the results herein presented.

#### 2.4.3. Antioxidant Potential Assays

The ferric reducing antioxidant power (FRAP), scavenging of DPPH, and ABTS free radical assays were used to evaluate the antioxidant capacity of *Galium* species (Table 1). These methods are simple and widely used for the evaluation of antioxidant capacity of herbal extracts/pure compounds. Moreover, the values regarding the total phenolic content (TPC) and total flavonoid content (TFC) are in accordance with antioxidant capacity values of the extracts. In the DPPH assay, *G. purpureum* (6.3 ± 0.7 mg TE/g extract) exhibited a higher DPPH scavenging capacity than any other considered species (0.4–1.9 mg TE/g extract). The ABTS value for *G. purpureum* (16.7 ± 0.8 mg TE/g extract) was higher in comparison with the values obtained for the other considered species, which ranged from 4.5 to 7.6 mg TE/g extract.

#### 2.4.4. Tyrosinase Inhibitory Activity

*Galium* extracts had good tyrosinase inhibitory activities (4.66–70.98% at 8 mg/mL), as reported in Table 1. The extract of *G. album* presented the highest tyrosinase inhibition, with a value of 70.98%. Despite the highest concentration of rutin and chlorogenic acid, the ethanolic extract of *G. rivale* showed no inhibitory effect against tyrosinase. This shows that the synergic effect of the compounds from the tested *Galium* samples have no or low inhibitory effects in some cases, although it was demonstrated by many studies that phenolic and flavonoid compounds are, in general, good inhibitors of tyrosinase [19]. The modest tyrosinase inhibitory activity for *Galium* species is confirmed by other studies as well. For example, Chiocchio et al. reported no tyrosinase inhibitory activity for *G. album* [35]. The low inhibitory activity of these extracts can be explained by the presence of other nondetected compounds, which might block or interfere with the enzyme.

### 2.5. Quantitative Analysis of Galium Species

The dry extracts were analyzed to establish the fingerprint of phenolic compounds in five *Galium* species. Table 2 summarizes the results obtained by means of a validated HPLC-PDA method for phenolics determination. All measurements were performed in triplicate in order to obtain standard deviation. It can be observed that the amount of the phenolic compounds for all *Galium* species is in the range from 2526.2–11345.1 μg g^−1^. The major biologically active compounds are chlorogenic acid and rutin. The highest number of the detected phenolic compounds was found in *G. rivale* (11345.1 μg g^−1^), where the main compound was chlorogenic acid (10192 ± 34 μg g^−1^), but the fingerprint was poorer in comparison with other species. The richest multicomponent pattern was observed in *G. pseudoaristatum*, but the quantity of phenolic compounds was the lowest (2526.2 μg g^−1^ ± 46.21). Chromatograms for each *Galium* sp. were reported in Supplementary Materials section S2.

*p*-OH benzoic acid, vanillic acid, epicatechin, syringic acid, 3-OH-4-MeO benzaldehyde, *p*-coumaric acid, *t*-ferulic acid, naringin, 2.3-diMeO benzoic acid, benzoic acid, *o*-coumaric acid, harpagoside, *t*-cinnamic acid, and naringenin were not reported into the table because they were not detected by the HPLC-PDA method.

## 3. Materials and Methods

### 3.1. Materials

Chemical standards of phenolic compounds (benzoic acid, carvacrol, catechin, chlorogenic acid, *t*-cinnamic acid, 8-cinnamoyl harpagide (harpagoside), *o*-coumaric acid, *p*-coumaric acid, 2,3-dimethoxybenzoic acid, epicatechin, *t*-ferulic acid, gallic acid, 3-hydroxybenzoic acid, 4-hydroxybenzoic acid, 3-hydroxy-4-methoxybenzaldehyde, naringin, naringenin, quercetin, rutin, sinapinic acid, syringic acid, vanillic acid (all purity > 98%)), *β*-cyclodextrin (≥97%), *n*-hexane (HPLC-grade), diethyl ether (≥99%), and chloroform (HPLC-grade) were purchased from Sigma-Aldrich (Milan, Italy).

Ethyl acetate (≥99%), acetonitrile (HPLC-grade), methanol (HPLC-grade), ethanol (HPLC-grade), acetic acid (≥99%) as well as d-(+)-glucose were obtained from Carlo Erba Reagents (Milan, Italy). Sodium chloride (≥99%) was obtained from Honeywell (Seelze, Germany). NADES (glycolic acid/betaine mixture) was newly synthesized and supplied by University of Perugia. It was chosen between differently structured novel DES and NADES mixtures for its suitable properties (low freezing point and low viscosity, absence of aromatic compounds in its composition, low cost and natural source of the molecules forming it). Ultra-pure water was obtained using a Millipore Milli-Q Plus water treatment system (18 MΩ cm at 23 °C, Millipore Bedford Corp., Bedford, MA, USA).

### 3.2. Sampling and Sample Preparation

Samples of *Galium* species were collected from different locations from Romania, as follows: *G. verum* L. from Apuseni mountains region, Sartăș, Alba County, Transylvania, Romania in June 2017, *G. album* Mill. from Podeni, Cluj Coutry, Romania and from Rimetea, Alba Coutry, Romania, *G. purpureum* L. and *G. pseudoaristatum* Schur from Băile Herculane, Caraş-Severin Coutry, in August 2014. All species were authenticated by Dr. Sabin Bădărău and Dr. Andrei Mocan, and voucher specimens were deposited at the herbarium of the Department of Pharmaceutical Botany, “Iuliu Hațieganu” University of Medicine and Pharmacy. Fresh herbal material was dried at room temperature until reaching a constant mass. Afterwards, the plant material was ground into a fine powder using a laboratory mill, mixed to obtain homogenous sample, and kept at 4 °C, for further analyses. All assays were carried out three times (three separate samplings) and in triplicate, and the values reported are represented by average and the standard deviation (S.D.).

### 3.3. Apparatus

#### 3.3.1. HPLC Analysis

The quantitative analysis of phenolic compounds was performed according to the reported method [36]. The chromatographic system consisted of HPLC Waters liquid chromatograph instrument (model 600 solvent pump, 2996 PDA). Mobile phase was directly on-line degassed by using a Biotech 4CH DEGASI Compact (Onsala, Sweden). For separation of phenolic compounds, C18 reversed-phase column (Prodigy ODS(3), 4.6 × 150 mm, 5 µm; Phenomenex, Torrance, CA), thermostated at 30 °C (±1 °C) was used. The collection and analysis of the data were performed by Empower v.2 software (Waters Corporation, Milford, MA, USA). The mobile phase was a mixture of solution A (3% solution of acetic acid in water) and solution B (3% solution of acetic acid in acetonitrile) in a ratio 93:7 and the gradient mode was applied. The total separation was completed in 1 h (the chemical standards chromatograms, retention times and maximum wavelengths are shown in *Supplementary Materials* section S3).

#### 3.3.2. Auxiliary Equipment

As auxiliary equipment for the extraction procedures, centrifuge model NF048 (Nuve, Ankara, Turkey), vortex (VELP Scientifica Srl, Usmate, Italy), and ultrasonic bath (Falc Instruments, Treviglio, Italy) were used. MAE was performed using an automatic Biotage Initiator™ 2.0 (Uppsala, Sweden) characterized by 2.45 GHz high-frequency microwaves and power range 0–300 W. An IR sensor probe controlled the internal vial temperature.

### 3.4. Extraction Procedures

**Figure 3:**
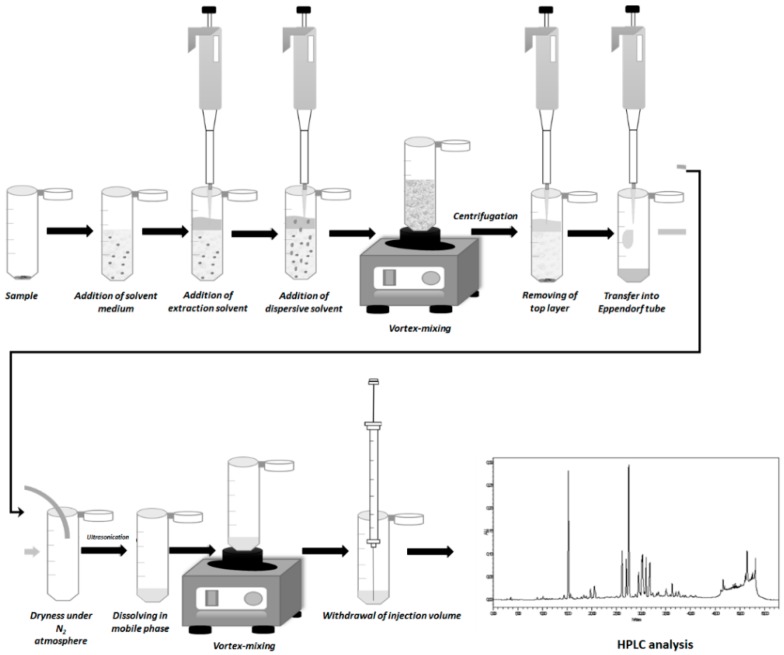
General extraction procedure.

Extraction optimization was carried out using *G. verum* and after, under optimized conditions, the microextraction procedure was applied for the other four *Galium* species. The following microextraction procedure were investigated: DLLME, ultrasound-assisted dispersive liquid-liquid microextraction (UA-DLLME), Salting-out liquid-liquid extraction (SA-LLE), and Sugaring-out liquid-liquid extraction (SULLE). The general procedure for the extractions reported in the following paragraphs was described in Figure 3.

#### 3.4.1. DLLME and UA-DLLME

10 mg of the dry plant material of *G. verum* were accurately weighted and placed into a 2 mL Eppendorf tube. Subsequently, 700 μL of solvent medium (water, 10% NaCl, NADES, IL or 1% β-cyclodextrin (β-CD)), 400 μL of ethyl acetate, and 300 μL of ethanol were added to the Eppendorf tube by automatic pipette. The solution was vortexed during 30 s until a cloudy solution was formed. In the case of UA-DLLME, after those steps, the test tube was placed into the ultrasound bath for 5 min. Then, the solution was kept at rest for 1 min, for the analytes to distribute into the extraction solvent. For the phase separation, the solution was centrifuged at 12000× *g* for 5 min. The extraction solvent was found on the top of the Eppendorf tube, and its whole volume was collected using a microsyringe and transferred to the new Eppendorf tube, and then dried under a gentle stream of nitrogen. The dried residue was redissolved in 50 μL of mobile phase under ultrasonication for 5 min and 20 μL of the obtained solution were injected into the HPLC system.

#### 3.4.2. Salting-Out-LLE

For salting-out-DLLME (SA-LLE), 10 mg of the dry herbal material of *G. verum* were placed into a 2 mL Eppendorf tube. Then, 200 μL of water and 400 μL of acetonitrile were added. To obtain the phase separation, 200 μL of 300 g L^−1^ solution of NaCl were rapidly injected into the Eppendorf tube. The mixture was vortexed for 1 min and a cloudy solution was formed. The next procedures were the same as in the Section 3.4.1.

#### 3.4.3. Sugaring-Out-LLE

For the sugaring-out-LLE (SULLE), the procedure was similar to the SA-LLE. Instead of aqueous NaCl, 200 μL of glucose solution (600 g L^−1^) was utilized for phase separation.

#### 3.4.4. MAE

10 mg of the dry plant material were placed into a 2 mL sealed vessel suitable for an automatic single-mode microwave reactor and 1 mL of appropriate solvent medium (see Section 3.3.) was added, forming a yellow-green emulsion. MAE was carried out heating by microwave irradiation for 13 min 8 s at 80 °C (which correspond approximatively to 24 h of maceration at 25 °C), and then cooling to room temperature by pressurized air. Then, the homogenate was centrifuged at 12000× *g* for 5 min and 20 μL of the solution were directly injected into the HPLC system.

### 3.5. Total Phenolic and Flavonoid Content, Antioxidant Capacity, and Tyrosinase Inhibitory Activity

#### 3.5.1. Antioxidant Assays

##### Total Phenolic Content (TPC) by Spectrophotometric Assay

The TPC was determined using the Folin–Ciocâlteu method described by Mocan et al. [37]. For a high throughput of samples, a SPECTROstar Nano Multi—Detection Microplate Reader with 96-well plates (BMG Labtech, Ortenberg, Germany) was used. Briefly, a mixture solution consisting of 20 µL of extract, 100 µL of Folin-Ciocâlteu reagent, and 80 µL of sodium carbonate (Na_2_CO_3_, 7.5% *w*/*v*) was homogenized and incubated at room temperature in the dark for 30 min. Afterwards, the absorbance of the samples was measured at 760 nm. Gallic acid was used as a reference standard, and the TPC was expressed as gallic acid equivalents (GAE) in mg/g dry weight (d.w.) of plant material.

##### Total Flavonoid Content (TFC) by Spectrophotometric Assay

The total flavonoid content (TFC) was calculated and expressed as quercetin equivalents using a method previously described by Mocan et al. [38]. Briefly, a 100 µL aliquot of 2% AlCl_3_ aqueous solution was mixed with 100 µL of sample. After an incubation time of 15 min, the absorbance of the sample was measured at 420 nm. Quercetin was used as a reference standard, and the TFC was expressed as quercetin equivalents (QE) in mg/g dry weight (d.w.) of plant material.

##### DPPH Radical Scavenging Assay

The capacity to scavenge the “stable” free radical DPPH, monitored according to the method described by Martins et al. [39], with some modifications, was performed by using a SPECTROstar Nano microplate reader (BMG Labtech, Offenburg, Germany). The reaction mixture in each of the 96-wells consisted of 30 µL of sample solution (in an appropriated dilution) and a 0.004% methanolic solution of DPPH. The mixture was further incubated for 30 min in the dark, and the reduction of the DPPH radical was determined at 515 nm. Trolox was used as a standard reference and the results were expressed as Trolox equivalents per g of dry weight herbal extract (mg TE/g d.w. of herbal extract).

##### Trolox Equivalent Antioxidant Capacity (TEAC) Assay

In the Trolox equivalent antioxidant capacity (TEAC) assay, the antioxidant capacity is reflected in the ability of the *Galium* extracts to decrease the color, reacting directly with the ABTS radical. The latter was obtained by oxidation of ABTS (2,2’-azinobis(3-ethylbenzothiazoline-6-sulfonic acid)) with potassium peroxydisulfate (K_2_S_2_O_8_). The amount of ABTS radical consumed by the tested compound was measured at 760 nm after 6 min of reaction time. The evaluation of the antioxidant capacity was obtained using the total change in absorbance at this wavelength. The percentage of ABTS consumption was transformed in Trolox equivalents (TE) using a calibration curve.

##### FRAP Assay

In FRAP assays, the reduction of Fe^3+^-TPTZ to blue-colored Fe^2+^-TPTZ complex was monitored by the method described by Damiano et al., (2017) with slight modifications [40]. The FRAP reagent was prepared by mixing ten volumes of acetate buffer (300 mM, pH 3.6), one volume of TPTZ solution (10 mM TPTZ in 40 mM HCl) and one volume of FeCl_3_ solution (20 mM FeCl_3_·6H_2_O in 40 mM HCl). Reaction mixture (25 µL sample and 175 µL FRAP reagent) was incubated in the dark for 30 min at room temperature and the absorbance of each solution was measured at 593 nm using a SPECTROstar Nano Multi-Detection Microplate Reader with 96-well plates (BMG Labtech, Ortenberg, Germany). A Trolox^TM^ calibration curve (0.01–0.10 mg/mL) was plotted as a function of blue-colored Fe^2+^-TPTZ complex formation, and the results were expressed as milligrams of trolox equivalents (TE) per milligram of extract (mg TE/mg extract).

#### 3.5.2. Tyrosinase Inhibitory Activity

Tyrosinase inhibitory activity of each sample was determined by the method previously described by Likhitwitayawuid and Sritularak, (2001) and Masuda *et al*., (2005) [41,42] using a SPECTROstar Nano Multi-Detection Microplate Reader with 96-well plates (BMG Labtech, Ortenberg, Germany). Samples were dissolved in water containing 5% DMSO; for each sample four wells were designated as A, B, C, D; each one contained the reaction mixture (200 µL) as follows: (A) 120 µL of 0.66 M phosphate buffer solution (pH = 6.8) (PBS) and 40 µL of mushroom tyrosinase in PBS (46 U/mL) (Tyr), (B) 160 µL of PBS, (C) 80 µL of PBS, 40 µL of Tyr, and 40 µL of sample, and (D) 120 µL of PBS and 40 µL of sample. The plate was then incubated at room temperature for 10 min; after incubation, 40 µL of 2.5 mM L-DOPA in PBS solution were added in each well and the mixtures were incubated again at room temperature for 20 min. The absorbance of each well was measured at 475 nm, and the inhibition percentage of the tyrosinase activity was calculated by the following equation, using as positive control a kojic acid solution (0.10 mg/mL):

(1) $$\%I=\frac{\left(A-B\right)-\left(C-D\right)}{\left(A-B\right)}\times 100$$

The results were also expressed as mg kojic acid equivalents per gram of dry weight extract (mg KAE/g extract) using a calibration curve between 0.01–0.10 mg kojic acid per milliliter of solution.

### 3.6. Statistical Analysis

All experiments were performed in triplicate and the results were expressed as the mean value ± standard deviation (S.D.). All comparisons were determined by using two-way ANOVA followed by Bonferroni post-test and GraphPad Prism v.4 for data elaboration. Raw data regarding the statistical analyses were reported in *Supplementary Materials* section S1.

## 4. Conclusions

In this work, a microextraction procedure was developed and applied for the establishment of the multicomponent phenolic pattern of aerial parts from *G. verum*, *G. album*, *G. rivale*, *G. pseudoaristatum*, and *G. purpureum*. The DLLME procedure in NADES solvent medium could provide high extraction efficiency within a short extraction time and with good correspondence with the MAE procedure. The biological results showed that *G. purpureum* and *G. verum* extracts contained the highest total phenolic and flavonoid contents, respectively. *G. purpureum* extract was the most active extract in terms of antioxidant capacity, whereas the *G. album* extract exhibited the strongest inhibitory effect against tyrosinase, an enzyme involved in several skin disorders. The results indicate that *Galium* extracts have the potential to be used as an alternative source of multifunctional agents and are a promising starting point for development of new bioactive formulations. Further studies are essential for the isolation of pure bioactive compounds and investigation of their molecular mechanisms of action.

## Figures and Tables

**Table 1:** Total phenolic and flavonoid content, antioxidant capacity, and enzyme inhibitory effects of the extracts of *Galium* spp. (values expressed are means ± S.D. of three measurements).

	TPC (mg GAE/g Extract)	TFC (mg QE/g Extract)	DPPH Scavenging (mg TE/g Extract)	ABTS Scavenging (mg TE/g Extract)	FRAP (mg TE/g Extract)	Tyrosinase Inhibit. (mg KAE/g Extract)	Tyrosinase Inhibit. (% Inhibition)
***G. verum***	3.1 ± 0.3 ^a,e^	8.60 ± 0.07 ^a,e^	1.9 ± 0.5 ^a,e^	6.15 ± 0.02 ^a,e^	21.9 ± 0.9 ^a,c,e^	7.3 ± 0.2 ^a,e^	36.96
***G. album***	2.7 ± 0.1 ^a,e^	4.88 ± 0.03 ^a,e^	1.1 ± 0.2 ^a,e^	6.1 ± 0.1 ^a,e^	17.19 ± 0.04 ^b,e^	13.8 ± 3.4 ^e^	70.98
***G. rivale***	1.3 ± 0.2 ^a,d,e^	4.01 ± 0.08 ^a,c,e^	0.4 ± 0.25 ^a,e^	4.5 ± 0.5 ^a,d,e^	12.6 ± 0.2 ^a,e^	na	na
***G. pseudoaristatum***	4.5 ± 0.6 ^a,d,e^	6.7 ± 0.3 ^a,c^	1.9 ± 0.1 ^a,e^	7.6 ± 0.2 ^a,d,e^	19.4 ± 0.9 ^a,b,c,e^	2.5 ± 1.6 ^e^	4.66
***G. purpureum***	10.3 ± 0.8 ^e^	8.50 ± 0.04 ^a,e^	6.3 ± 0.7 ^e^	16.7 ± 0.8 ^e^	45.2 ± 1.1 ^e^	6.3 ± 1.4 ^a,e^	29.71
**KA (0.1 mg/mL)**							62.52

Na—not active; for tyrosinase inhibition (in percentages), the extracts concentration was 8 mg/mL. TFC = Total Flavonoid Content by spectrophotometric assay; TPC = Total Phenolic Content by spectrophotometric assay; ABTS = 2,2’-azino-bis(3-ethylbenzothiazoline-6-sulphonic acid); DPPH = 2,2-diphenyl-1-picryl-hydrazyl-hydrate; FRAP = Ferric Reducing Antioxidant Power; GAE = Gallic Acid Equivalents; QE = Quercetin Equivalents; TE = Trolox Equivalents; KAE = Kojic Acid Equivalents; KA = Kojic Acid. Raw data regarding the statistical analyses were reported in *Supplementary Materials* section S1. Data marked with different letters indicates significant difference (*p* < 0.05).

**Table 2:** Phenolic compounds quantified in Galium spp. using DLLME in 15% NADES (values expressed are means ± S.D. of three measurements).

Metabolite	*Galium* Species				
	*G. verum*	*G. album*	*G. rivale*	*G. pseudoaristatum*	*G. purpureum*
	Conc., μg/g				
	Mean (±S.D.)	Mean (±S.D.)	Mean (±S.D.)	Mean (±S.D.)	Mean (±S.D.)
**Gallic acid**				109 (±7) ^a,b,e^	23.2 (±1.5) ^a,b,e^
**Catechin**		380 (±96) ^a,e^	63.5 (±2.5) ^a,e^	203 (±30) ^a,e^	
**Chlorogenic acid**	2986 (±75) ^e^	8310 (±231) ^e^	10192 (±34) ^e^	1640 (±30) ^e^	5572 (±205) ^e^
**3-OH benzoic acid**	853 (±184) ^e^			87.4 (±12.6) ^a,c^	374 (±16) ^b,c,d,e^
**Rutin**	3624 (±97) ^e^	275 (±38) ^a,c,e^	987 (±24) ^e^	283 (±5) ^a,b,c,d,e^	137 (±13) ^a,d,e^
**Sinapinic acid**				55.7 (±0.9) ^a,d,e^	203 (±81) ^a,b^
**Quercetin**	89.6 (±15.5) ^a,e^	84.1 (±8.3) ^a,c,e^		67.9 (±11.6) ^a,c,e^	
**Carvacrol**	101 (±2) ^a,e^	84.1 (±0.9) ^a,c,e^	102.6 (±0.9) ^a,e^	81.8 (±2.1) ^a,c,e^	162 (±26) ^a,c,e^
**Total μg/g**	**7654 (±222)**	**9133 (±253)**	**11345 (±42)**	**2526 (±47)**	**6471 (±223)**

Data marked with different letters indicate significant difference (*p* < 0.05).

## Supplementary Materials

The following are available online. In section S1 were reported all the raw data obtained using GraphPad v.4 and Bonferroni post-test related to the statistical analyses for Figures and Tables present in the main text. In section S2 the chromatograms of dry extracts of Galium species obtained using DLLME (@ 278 nm as example of wavelength in which all compounds show absorbance) were reported. In section S3 the chromatogram (@ 278 nm as example of wavelength in which all compounds show absorbance) for the 22 chemical standards, with a table reporting the retention times and the maximum wavelengths used for the quantitative analyses was reported.

## Abbreviations

ABTS	2,2’-azino-bis(3-ethylbenzothiazoline-6-sulphonic acid)
β-CD	β-cyclodextrin
DLLME	Dispersive Liquid–Liquid MicroExtraction
DMSO	Dimethyl sulfoxide
DPPH	2,2-diphenyl-1-picryl-hydrazyl-hydrate
d.w.	dry weight
FRAP	Ferric Reducing Antioxidant Power
GAE	Gallic Acid Equivalents
GC	Gas Chromatography
HPLC	High Performance Liquid Chromatography
HPLC-PDA	High Performance Liquid Chromatography-Photodiode array detector
ILs	ionic liquids
KA	Kojic Acid
KAE	Kojic Acid Equivalents
LPME	Liquid Phase MicroExtraction
MAE	Microwave-Assisted Extraction
NADES	NAtural Deep Eutectic Solvent
QE	Quercetin Equivalents
SA-LLE	Salting-out Liquid-liquid extraction
SULLE	Sugaring-out Liquid-liquid extraction
TCPC	Total Concentration of Phenolic Compounds
TE	Trolox Equivalents
TEAC	Trolox Equivalent Antioxidant Capacity
TFC	Total Flavonoid Content by spectrophotometric assay
TPC	Total Phenolic Content by spectrophotometric assay
UA-DLLME	Ultrasound-Assisted Dispersive Liquid–Liquid MicroExtraction
