# Supplementary material for: Liquid Phase and Microwave-Assisted Extractions for Multicomponent Phenolic Pattern Determination of Five Romanian Galium Species Coupled with Bioassays
Source: Molecules. 2019 Mar 28;24(7):1226. doi: 10.3390/molecules24071226 (PMC6480365; doi:10.3390/molecules24071226)
Supplement: Supplementary file 1 [file molecules-24-01226-s001.pdf]

Liquid Phase and Microwave-Assisted Extractions for Multicomponent Phenolic Pattern Determination of Five Romanian *Galium* Species Coupled with Bioassays

Andrei Mocan [1], Alina Diuzheva [2], Sabin Bădărău [3], Cadmiel Moldovan [1], Vasil Andruch [2], Simone Carradori [4], Cristina Campestre [4], Angela Tartaglia [4], Marta De Simone [4], Dan Vodnar [5], Matteo Tiecco [6], Raimondo Germani [6], Gianina Crișan [1] and Marcello Locatelli [4,*]

[1] Department of Pharmaceutical Botany, "Iuliu Hațieganu" University of Medicine and Pharmacy, 400012 Cluj-Napoca, Romania; mocan.andrei@umfcluj.ro (A.M.); moldovan.cadmiel@yahoo.com (C.M.); gcrisan@umfcluj.ro (G.C.)

[2] Department of Analytical Chemistry, Pavol Jozef Šafárik University, SK-04180 Košice, Slovakia; adyuzheva@gmail.com (A.D.); vasil.andruch@gmail.com (V.A.)

[3] Department of Environmental Sciences, Babeș-Bolyai University, 400084 Cluj-Napoca, Romania; alexandru@transsilvanica.net

[4] Department of Pharmacy, University "G. D'Annunzio" of Chieti-Pescara, 66100 Chieti, Italy; simone.carradori@unich.it (S.C.); cristina.campestre@unich.it (C.C.); angela.tartaglia@unich.it (A.T.); marta.desimone@studenti.unich.it (M.D.S.)

[5] Department of Food Science, University of Agricultural Sciences and Veterinary Medicine, 400372 Cluj-Napoca, Romania; dan.vodnar@usamvcluj.ro

[6] Department of Chemistry, Biology and Biotechnology, University of Perugia, 06132 Perugia, Italy; matteotiecco@gmail.com (M.T.); raimondo.germani@unipg.it (R.G.)

* Correspondence: m.locatelli@unich.it; Tel.: +39-0871-3554590; Fax: +39-0871-3554911

SUPPLEMENTARY MATERIALS

In **section S1** were reported all the raw data obtained using GraphPad v.4 and Bonferroni post-test related to the statistical analyses for Figures ad Tables present in the main text.

In **section S2** the chromatograms of dry extracts of *Galium* species obtained using DLLME (@ 278 nm as example of wavelength in which all compounds show absorbance) were reported.

In **section S3** the chromatogram (@ 278 nm as example of wavelength in which all compounds show absorbance) for the 22 chemical standards, with a table reporting the retention times and the maximum wavelengths used for the quantitative analyses was reported.

Section S1

Raw data obtained using GraphPad v.4 and Bonferroni post-test related to the statistical analyses for Figures ad Tables present in the main text.

Table Analyzed	**Figure 1**			
Two-way ANOVA				
Source of Variation	% of total variation	P value		
Interaction	54,43	P<0.0001		
procedure	23,69	P<0.0001		
extraction medium	20,78	P<0.0001		
Source of Variation	P value summary	Significant?		
Interaction	***	Yes		
procedure	***	Yes		
extraction medium	***	Yes		
Source of Variation	Df	Sum-of-squares	Mean square	F
Interaction	10	3876000000	387600000	178,7
procedure	2	1686000000	843200000	388,7
extraction medium	5	1480000000	296000000	136,4
Residual	36	78090000	2169000	
Number of missing values	-18			
Bonferroni posttests				
Water vs. DLLME				
extraction medium	Water	DLLME	Difference	95% CI of diff.
Water	17900	0	-17900	-21760 to -14040
NaCl	0	28090	28090	24230 to 31950
b-cyclodextrin	0	17650	17650	13790 to 21510
NADES	0	30970	30970	27110 to 34830
SA-LLE	0	12510	12510	8651 to 16380
SULLE	0	10210	10210	6352 to 14080
extraction medium	Difference	t	P value	Summary
Water	-17900	14,88	P<0.001	***
NaCl	28090	23,36	P<0.001	***
b-cyclodextrin	17650	14,68	P<0.001	***
NADES	30970	25,75	P<0.001	***
SA-LLE	12510	10,41	P<0.001	***

SULLE	10210	8,493	P<0.001	***

Water vs. UA-DLLME

extraction medium	Water	UA-DLLME	Difference	95% CI of diff.
Water	17900	0	-17900	-21760 to -14040
NaCl	0	22930	22930	19070 to 26800
b-cyclodextrin	0	27170	27170	23300 to 31030
NADES	0	0	0	-3862 to 3862
SA-LLE	0	0	0	-3862 to 3862
SULLE	0	0	0	-3862 to 3862

extraction medium	Difference	t	P value	Summary
Water	-17900	14,88	P<0.001	***
NaCl	22930	19,07	P<0.001	***
b-cyclodextrin	27170	22,59	P<0.001	***
NADES	0	0	P > 0.05	ns
SA-LLE	0	0	P > 0.05	ns
SULLE	0	0	P > 0.05	ns

DLLME vs. UA-DLLME

extraction medium	DLLME	UA-DLLME	Difference	95% CI of diff.
Water	0	0	0	-3862 to 3862
NaCl	28090	22930	-5154	-9017 to -1292
b-cyclodextrin	17650	27170	9517	5655 to 13380
NADES	30970	0	-30970	-34830 to -27110
SA-LLE	12510	0	-12510	-16380 to -8651
SULLE	10210	0	-10210	-14080 to -6352

extraction medium	Difference	t	P value	Summary
Water	0	0	P > 0.05	ns
NaCl	-5154	4,286	P<0.001	***
b-cyclodextrin	9517	7,914	P<0.001	***
NADES	-30970	25,75	P<0.001	***
SA-LLE	-12510	10,41	P<0.001	***
SULLE	-10210	8,493	P<0.001	***

Table Analyzed **Figure 2a**

Two-way ANOVA

Source of Variation	% of total variation	P value
Interaction	60,96	P<0.0001
procedure	35	P<0.0001
medium concentration	2,18	P<0.0001

Source of Variation	P value summary	Significant?
Interaction	***	Yes
procedure	***	Yes
medium concentration	***	Yes

Source of Variation	Df	Sum-of-squares	Mean square	F
Interaction	16	9013000000	563300000	110,7
procedure	2	5176000000	2588000000	508,4
medium concentration	8	322300000	40280000	7,914
Residual	54	274900000	5090000	

Number of missing values	-27

Bonferroni posttests

DLLME vs. UA-DLLME

medium concentration	DLLME	UA-DLLME	Difference	95% CI of diff.
b-CD 0.5%	0	24530	24530	18500 to 30560
b-CD 1.0%	0	27170	27170	21130 to 33200
b-CD 1.5%	0	21910	21910	15880 to 27940
NaCl 5%	18770	0	-18770	-24800 to -12730
NaCl 10%	28090	0	-28090	-34120 to -22060
NaCl 15%	28520	0	-28520	-34550 to -22490
NADES 5%	30270	0	-30270	-36310 to -24240
NADES 10%	30970	0	-30970	-37000 to -24940
NADES 15%	30460	0	-30460	-36490 to -24430

medium concentration	Difference	t	P value	Summary
b-CD 0.5%	24530	13,32	P<0.001	***
b-CD 1.0%	27170	14,75	P<0.001	***
b-CD 1.5%	21910	11,89	P<0.001	***
NaCl 5%	-18770	10,19	P<0.001	***
NaCl 10%	-28090	15,25	P<0.001	***
NaCl 15%	-28520	15,48	P<0.001	***
NADES 5%	-30270	16,43	P<0.001	***
NADES 10%	-30970	16,81	P<0.001	***
NADES 15%	-30460	16,53	P<0.001	***

DLLME vs. MAE

medium concentration	DLLME	MAE	Difference	95% CI of diff.
b-CD 0.5%	0	31720	31720	25690 to 37750
b-CD 1.0%	0	23580	23580	17550 to 29610
b-CD 1.5%	0	28490	28490	22460 to 34520
NaCl 5%	18770	30000	11230	5202 to 17270
NaCl 10%	28090	31850	3765	-2266 to 9797
NaCl 15%	28520	15090	-13430	-19460 to -7394
NADES 5%	30270	29660	-613,3	-6645 to 5418
NADES 10%	30970	27320	-3644	-9676 to 2388
NADES 15%	30460	32000	1537	-4495 to 7568

medium concentration	Difference	t	P value	Summary
b-CD 0.5%	31720	17,22	P<0.001	***
b-CD 1.0%	23580	12,8	P<0.001	***
b-CD 1.5%	28490	15,47	P<0.001	***
NaCl 5%	11230	6,098	P<0.001	***
NaCl 10%	3765	2,044	P > 0.05	ns
NaCl 15%	-13430	7,288	P<0.001	***
NADES 5%	-613,3	0,3329	P > 0.05	ns
NADES 10%	-3644	1,978	P > 0.05	ns
NADES 15%	1537	0,8341	P > 0.05	ns

UA-DLLME vs. MAE

medium concentration	UA-DLLME	MAE	Difference	95% CI of diff.
b-CD 0.5%	24530	31720	7191	1160 to 13220
b-CD 1.0%	27170	23580	-3588	-9619 to 2444
b-CD 1.5%	21910	28490	6581	548.9 to 12610
NaCl 5%	0	30000	30000	23970 to 36030
NaCl 10%	0	31850	31850	25820 to 37890
NaCl 15%	0	15090	15090	9060 to 21120
NADES 5%	0	29660	29660	23630 to 35690
NADES 10%	0	27320	27320	21290 to 33360
NADES 15%	0	32000	32000	25960 to 38030

medium concentration	Difference	t	P value	Summary
b-CD 0.5%	7191	3,904	P<0.01	**
b-CD 1.0%	-3588	1,948	P > 0.05	ns
b-CD 1.5%	6581	3,572	P<0.01	**
NaCl 5%	30000	16,29	P<0.001	***
NaCl 10%	31850	17,29	P<0.001	***
NaCl 15%	15090	8,192	P<0.001	***
NADES 5%	29660	16,1	P<0.001	***
NADES 10%	27320	14,83	P<0.001	***
NADES 15%	32000	17,37	P<0.001	***

Table Analyzed **Figure 2b**

Two-way ANOVA

Source of Variation	% of total variation	P value
Interaction	21,67	P<0.0001
additives	16,47	P<0.0001
ratio	56,38	P<0.0001

Source of Variation	P value summary	Significant?
Interaction	***	Yes
additives	***	Yes
ratio	***	Yes

Source of Variation	Df	Sum-of-squares	Mean square	F
Interaction	4	518500000	129600000	17,79
additives	2	394000000	197000000	27,04
ratio	2	1349000000	674400000	92,55
Residual	18	131200000	7287000	

Number of missing values -9

Bonferroni posttests

NaCl vs. NADES

ratio	NaCl	NADES	Difference	95% CI of diff.
5:1.4	20150	16030	-4120	-11060 to 2820
10:1.4	28090	45910	17820	10880 to 24760
15:1.4	20150	30460	10310	3372 to 17250

ratio	Difference	t	P value	Summary
5:1.4	-4120	1,869	P > 0.05	ns
10:1.4	17820	8,084	P<0.001	***
15:1.4	10310	4,679	P<0.001	***

NaCl vs. b-CD

ratio	NaCl	b-CD	Difference	95% CI of diff.
5:1.4	20150	13150	-6995	-13930 to - 55.00
10:1.4	28090	27170	-922	-7862 to 6018
15:1.4	20150	27470	7323	383.5 to 14260

ratio	Difference	t	P value	Summary
5:1.4	-6995	3,174	P < 0.05	*
10:1.4	-922	0,4183	P > 0.05	ns
15:1.4	7323	3,323	P < 0.05	*

NADES vs. b-CD

ratio	NADES	b-CD	Difference	95% CI of diff.
5:1.4	16030	13150	-2875	-9814 to 4065
10:1.4	45910	27170	-18740	-25680 to -11800
15:1.4	30460	27470	-2989	-9929 to 3951

ratio	Difference	t	P value	Summary
5:1.4	-2875	1,304	P > 0.05	ns
10:1.4	-18740	8,502	P<0.001	***
15:1.4	-2989	1,356	P > 0.05	ns

Table Analyzed **Figure 2c**

Two-way ANOVA

Source of Variation	% of total variation	P value
Interaction	2,08	0,0005
additives	1,9	P<0.0001
ratio	94,68	P<0.0001

Source of Variation	P value summary	Significant?
Interaction	***	Yes
additives	***	Yes
ratio	***	Yes

Source of Variation	Df	Sum-of-squares	Mean square	F
Interaction	6	96050000	16010000	6,2
additives	2	87620000	43810000	16,97
ratio	3	4375000000	1458000000	564,8
Residual	24	61970000	2582000	

Number of missing values -12

Bonferroni posttests

NaCl vs. NADES

ratio	NaCl	NADES	Difference	95% CI of diff.
Ethyl acetate	28090	30460	2371	-1784 to 6525
Diethyl ether	17580	13700	-3884	-8038 to 270.6
Chloroform	3604	7092	3488	-666.1 to 7642
Hexane	0	0	0	-4154 to 4154

ratio	Difference	t	P value	Summary

Ethyl acetate	2371	1,807	P > 0.05	ns
Diethyl ether	-3884	2,96	P < 0.05	*
Chloroform	3488	2,659	P > 0.05	ns
Hexane	0	0	P > 0.05	ns

NaCl vs. b-CD

ratio	NaCl	b-CD	Difference	95% CI of diff.
Ethyl acetate	28090	27170	-922	-5076 to 3232
Diethyl ether	17580	9122	-8458	-12610 to -4304
Chloroform	3604	845,3	-2759	-6913 to 1395
Hexane	0	0	0	-4154 to 4154

ratio	Difference	t	P value	Summary
Ethyl acetate	-922	0,7027	P > 0.05	ns
Diethyl ether	-8458	6,447	P<0.001	***
Chloroform	-2759	2,103	P > 0.05	ns
Hexane	0	0	P > 0.05	ns

NADES vs. b-CD

ratio	NADES	b-CD	Difference	95% CI of diff.
Ethyl acetate	30460	27170	-3293	-7447 to 861.6
Diethyl ether	13700	9122	-4575	-8729 to -420.5
Chloroform	7092	845,3	-6247	-10400 to -2093
Hexane	0	0	0	-4154 to 4154

ratio	Difference	t	P value	Summary
Ethyl acetate	-3293	2,51	P > 0.05	ns
Diethyl ether	-4575	3,487	P<0.01	**
Chloroform	-6247	4,761	P<0.001	***
Hexane	0	0	P > 0.05	ns

Table Analyzed **Figure 2d**

Two-way ANOVA

Source of Variation	% of total variation	P value
Interaction	11,27	0,017
additives	30,11	P<0.0001
ratio	45,96	P<0.0001

Source of Variation	P value summary	Significant?
Interaction	*	Yes
additives	***	Yes

ratio	***	Yes

Source of Variation	Df	Sum-of-squares	Mean square	F
Interaction	4	101900000	25470000	4,006
additives	2	272100000	136100000	21,41
ratio	2	415400000	207700000	32,68
Residual	18	114400000	6356000	

Number of missing values	-9

Bonferroni posttests

NaCl vs. NADES

ratio	NaCl	NADES	Difference	95% CI of diff.
300	24890	28090	3201	-3280 to 9683
400	31630	30460	-1170	-7651 to 5311
500	17000	27170	10170	3685 to 16650

ratio	Difference	t	P value	Summary
300	3201	1,555	P > 0.05	ns
400	-1170	0,5683	P > 0.05	ns
500	10170	4,939	P<0.001	***

NaCl vs. b-CD

ratio	NaCl	b-CD	Difference	95% CI of diff.
300	24890	20840	-4045	-10530 to 2436
400	31630	26230	-5402	-11880 to 1079
500	17000	15320	-1675	-8157 to 4806

ratio	Difference	t	P value	Summary
300	-4045	1,965	P > 0.05	ns
400	-5402	2,624	P > 0.05	ns
500	-1675	0,8138	P > 0.05	ns

NADES vs. b-CD

ratio	NADES	b-CD	Difference	95% CI of diff.
300	28090	20840	-7246	-13730 to -765.0
400	30460	26230	-4232	-10710 to 2249
500	27170	15320	-11840	-18320 to -5360

ratio	Difference	t	P value	Summary
300	-7246	3,52	P<0.01	**
400	-4232	2,056	P > 0.05	ns
500	-11840	5,753	P<0.001	***

Table Analyzed	**Figure 2e**	

Two-way ANOVA

Source of Variation	% of total variation	P value
Interaction	15,86	P<0.0001
additives	16,6	P<0.0001
ratio	63,19	P<0.0001

Source of Variation	P value summary	Significant?
Interaction	***	Yes
additives	***	Yes
ratio	***	Yes

Source of Variation	Df	Sum-of-squares	Mean square	F
Interaction	4	373400000	93340000	16,4
additives	2	390900000	195500000	34,35
ratio	2	1488000000	743800000	130,7
Residual	18	102400000	5691000	

Number of missing values	-9

Bonferroni posttests

NaCl vs. NADES

ratio	NaCl	NADES	Difference	95% CI of diff.
100	12740	14100	1361	-4771 to 7494
200	36610	25860	-10750	-16880 to -4613
300	28090	30460	2371	-3762 to 8504

ratio	Difference	t	P value	Summary
100	1361	0,699	P > 0.05	ns
200	-10750	5,517	P<0.001	***
300	2371	1,217	P > 0.05	ns

NaCl vs. b-CD

ratio	NaCl	b-CD	Difference	95% CI of diff.
100	12740	8133	-4604	-10740 to 1528
200	36610	15190	-21420	-27550 to -15290
300	28090	27170	-922	-7055 to 5211

ratio	Difference	t	P value	Summary
100	-4604	2,364	P > 0.05	ns

200	-21420	11	P<0.001	***
300	-922	0,4734	P > 0.05	ns

NADES vs. b-CD

ratio	NADES	b-CD	Difference	95% CI of diff.
100	14100	8133	-5966	-12100 to 166.9
200	25860	15190	-10680	-16810 to -4543
300	30460	27170	-3293	-9426 to 2840

ratio	Difference	t	P value	Summary
100	-5966	3,063	P < 0.05	*
200	-10680	5,481	P<0.001	***
300	-3293	1,691	P > 0.05	ns

Table Analyzed **Table 1 spp**

Two-way ANOVA

Source of Variation	% of total variation	P value
Interaction	20,84	P<0.0001
species	18,86	P<0.0001
analysis	59,68	P<0.0001

Source of Variation	P value summary	Significant?
Interaction	***	Yes
species	***	Yes
analysis	***	Yes

Source of Variation	Df	Sum-of-squares	Mean square	F
Interaction	20	1483	74,16	101
species	4	1343	335,7	457,2
analysis	5	4248	849,7	1157
Residual	60	44,06	0,7343	

Number of missing values -30

Bonferroni posttests

verum vs. album

analysis	verum	album	Difference	95% CI of diff.
TPC	3,1	2,7	-0,4	-2.862 to 2.062
TFC	8,6	4,88	-3,72	-6.182 to -1.258
DPPH	1,9	1,1	-0,8	-3.262 to 1.662
ABTS	6,15	6,1	-0,05	-2.512 to 2.412
FRAP	21,9	17,19	-4,71	-7.172 to -2.248

Tyrosinase	7,3	13,8	6,5	4.038 to 8.962

analysis	Difference	t	P value	Summary
TPC	-0,4	0,5717	P > 0.05	ns
TFC	-3,72	5,317	P<0.001	***
DPPH	-0,8	1,143	P > 0.05	ns
ABTS	-0,05	0,07146	P > 0.05	ns
FRAP	-4,71	6,732	P<0.001	***
Tyrosinase	6,5	9,29	P<0.001	***

verum vs. rivale

analysis	verum	rivale	Difference	95% CI of diff.
TPC	3,1	1,3	-1,8	-4.262 to 0.6620
TFC	8,6	4,01	-4,59	-7.052 to -2.128
DPPH	1,9	0,4	-1,5	-3.962 to 0.9620
ABTS	6,15	4,5	-1,65	-4.112 to 0.8120
FRAP	21,9	12,6	-9,3	-11.76 to -6.838
Tyrosinase	7,3	0	-7,3	-9.762 to -4.838

analysis	Difference	t	P value	Summary
TPC	-1,8	2,573	P > 0.05	ns
TFC	-4,59	6,56	P<0.001	***
DPPH	-1,5	2,144	P > 0.05	ns
ABTS	-1,65	2,358	P > 0.05	ns
FRAP	-9,3	13,29	P<0.001	***
Tyrosinase	-7,3	10,43	P<0.001	***

verum vs. pseudoaristatum

analysis	verum	pseudoaristatum	Difference	95% CI of diff.
TPC	3,1	4,5	1,4	-1.062 to 3.862
TFC	8,6	6,7	-1,9	-4.362 to 0.5620
DPPH	1,9	1,9	0	-2.462 to 2.462
ABTS	6,15	7,6	1,45	-1.012 to 3.912
FRAP	21,9	19,4	-2,5	-4.962 to -0.03799
Tyrosinase	7,3	2,5	-4,8	-7.262 to -2.338

analysis	Difference	t	P value	Summary
TPC	1,4	2,001	P > 0.05	ns
TFC	-1,9	2,716	P > 0.05	ns
DPPH	0	0	P > 0.05	ns
ABTS	1,45	2,072	P > 0.05	ns
FRAP	-2,5	3,573	P<0.01	**
Tyrosinase	-4,8	6,861	P<0.001	***

verum vs. purpureum

analysis	verum	purpureum	Difference	95% CI of diff.
TPC	3,1	10,3	7,2	4.738 to 9.662
TFC	8,6	8,5	-0,1	-2.562 to 2.362
DPPH	1,9	6,3	4,4	1.938 to 6.862
ABTS	6,15	16,7	10,55	8.088 to 13.01
FRAP	21,9	45,2	23,3	20.84 to 25.76
Tyrosinase	7,3	6,3	-1	-3.462 to 1.462

analysis	Difference	t	P value	Summary
TPC	7,2	10,29	P<0.001	***
TFC	-0,1	0,1429	P > 0.05	ns
DPPH	4,4	6,289	P<0.001	***
ABTS	10,55	15,08	P<0.001	***
FRAP	23,3	33,3	P<0.001	***
Tyrosinase	-1	1,429	P > 0.05	ns

album vs. rivale

analysis	album	rivale	Difference	95% CI of diff.
TPC	2,7	1,3	-1,4	-3.862 to 1.062
TFC	4,88	4,01	-0,87	-3.332 to 1.592
DPPH	1,1	0,4	-0,7	-3.162 to 1.762
ABTS	6,1	4,5	-1,6	-4.062 to 0.8620
FRAP	17,19	12,6	-4,59	-7.052 to -2.128
Tyrosinase	13,8	0	-13,8	-16.26 to -11.34

analysis	Difference	t	P value	Summary
TPC	-1,4	2,001	P > 0.05	ns
TFC	-0,87	1,243	P > 0.05	ns
DPPH	-0,7	1	P > 0.05	ns
ABTS	-1,6	2,287	P > 0.05	ns
FRAP	-4,59	6,56	P<0.001	***
Tyrosinase	-13,8	19,72	P<0.001	***

album vs. pseudoaristatum

analysis	album	pseudoaristatum	Difference	95% CI of diff.
TPC	2,7	4,5	1,8	-0.6620 to 4.262
TFC	4,88	6,7	1,82	-0.6420 to 4.282
DPPH	1,1	1,9	0,8	-1.662 to 3.262
ABTS	6,1	7,6	1,5	-0.9620 to 3.962
FRAP	17,19	19,4	2,21	-0.2520 to 4.672
Tyrosinase	13,8	2,5	-11,3	-13.76 to -8.838

analysis	Difference	t	P value	Summary
TPC	1,8	2,573	P > 0.05	ns
TFC	1,82	2,601	P > 0.05	ns
DPPH	0,8	1,143	P > 0.05	ns

ABTS	1,5	2,144	P > 0.05	ns
FRAP	2,21	3,159	P < 0.05	*
Tyrosinase	-11,3	16,15	P<0.001	***

album vs. purpureum

analysis	album	purpureum	Difference	95% CI of diff.
TPC	2,7	10,3	7,6	5.138 to 10.06
TFC	4,88	8,5	3,62	1.158 to 6.082
DPPH	1,1	6,3	5,2	2.738 to 7.662
ABTS	6,1	16,7	10,6	8.138 to 13.06
FRAP	17,19	45,2	28,01	25.55 to 30.47
Tyrosinase	13,8	6,3	-7,5	-9.962 to -5.038

analysis	Difference	t	P value	Summary
TPC	7,6	10,86	P<0.001	***
TFC	3,62	5,174	P<0.001	***
DPPH	5,2	7,432	P<0.001	***
ABTS	10,6	15,15	P<0.001	***
FRAP	28,01	40,03	P<0.001	***
Tyrosinase	-7,5	10,72	P<0.001	***

rivale vs. pseudoaristatum

analysis	rivale	pseudoaristatum	Difference	95% CI of diff.
TPC	1,3	4,5	3,2	0.7380 to 5.662
TFC	4,01	6,7	2,69	0.2280 to 5.152
DPPH	0,4	1,9	1,5	-0.9620 to 3.962
ABTS	4,5	7,6	3,1	0.6380 to 5.562
FRAP	12,6	19,4	6,8	4.338 to 9.262
Tyrosinase	0	2,5	2,5	0.03799 to 4.962

analysis	Difference	t	P value	Summary
TPC	3,2	4,574	P<0.001	***
TFC	2,69	3,845	P<0.01	**
DPPH	1,5	2,144	P > 0.05	ns
ABTS	3,1	4,431	P<0.001	***
FRAP	6,8	9,719	P<0.001	***
Tyrosinase	2,5	3,573	P<0.01	**

rivale vs. purpureum

analysis	rivale	purpureum	Difference	95% CI of diff.
TPC	1,3	10,3	9	6.538 to 11.46
TFC	4,01	8,5	4,49	2.028 to 6.952
DPPH	0,4	6,3	5,9	3.438 to 8.362
ABTS	4,5	16,7	12,2	9.738 to 14.66
FRAP	12,6	45,2	32,6	30.14 to 35.06
Tyrosinase	0	6,3	6,3	3.838 to 8.762

analysis	Difference	t	P value	Summary
TPC	9	12,86	P<0.001	***
TFC	4,49	6,417	P<0.001	***
DPPH	5,9	8,433	P<0.001	***
ABTS	12,2	17,44	P<0.001	***
FRAP	32,6	46,59	P<0.001	***
Tyrosinase	6,3	9,004	P<0.001	***

pseudoaristatum vs. purpureum

analysis	pseudoaristatum	purpureum	Difference	95% CI of diff.
TPC	4,5	10,3	5,8	3.338 to 8.262
TFC	6,7	8,5	1,8	-0.6620 to 4.262
DPPH	1,9	6,3	4,4	1.938 to 6.862
ABTS	7,6	16,7	9,1	6.638 to 11.56
FRAP	19,4	45,2	25,8	23.34 to 28.26
Tyrosinase	2,5	6,3	3,8	1.338 to 6.262

analysis	Difference	t	P value	Summary
TPC	5,8	8,29	P<0.001	***
TFC	1,8	2,573	P > 0.05	ns
DPPH	4,4	6,289	P<0.001	***
ABTS	9,1	13,01	P<0.001	***
FRAP	25,8	36,88	P<0.001	***
Tyrosinase	3,8	5,431	P<0.001	***

Table Analyzed **Table 2 spp**

Two-way ANOVA

Source of Variation	% of total variation	P value
Interaction	65,62	P<0.0001
species	2,74	P<0.0001
compound	31,58	P<0.0001

Source of Variation	P value summary	Significant?
Interaction	***	Yes
species	***	Yes
compound	***	Yes

Source of Variation	Df	Sum-of-squares	Mean square	F
Interaction	28	387200000	13830000	3318
species	4	16180000	4045000	970,5
compound	7	186400000	26620000	6387
Residual	80	333500	4168	

Number of missing values -40

Bonferroni posttests

verum vs. album

compound	verum	album	Difference	95% CI of diff.
Gallic	0	0	0	-187.7 to 187.7
Catechin	0	380,1	380,1	192.4 to 567.8
Chlorogenic	2986	8310	5324	5136 to 5512
3-OH	853	0	-853	-1041 to -665.3
Rutin	3624	275	-3349	-3537 to -3161
Sinapinic	0	0	0	-187.7 to 187.7
Quercetin	89,6	84,1	-5,5	-193.2 to 182.2
Carvacrol	101	84,07	-16,93	-204.7 to 170.8

compound	Difference	t	P value	Summary
Gallic	0	0	P > 0.05	ns
Catechin	380,1	7,211	P<0.001	***
Chlorogenic	5324	101	P<0.001	***
3-OH	-853	16,18	P<0.001	***
Rutin	-3349	63,53	P<0.001	***
Sinapinic	0	0	P > 0.05	ns
Quercetin	-5,5	0,1043	P > 0.05	ns
Carvacrol	-16,93	0,3212	P > 0.05	ns

verum vs. rivale

compound	verum	rivale	Difference	95% CI of diff.
Gallic	0	63,5	63,5	-124.2 to 251.2
Catechin	0	10190	10190	10000 to 10380
Chlorogenic	2986	0	-2986	-3174 to -2798
3-OH	853	987	134	-53.73 to 321.7
Rutin	3624	0	-3624	-3812 to -3436
Sinapinic	0	0	0	-187.7 to 187.7
Quercetin	89,6	0	-89,6	-277.3 to 98.13
Carvacrol	101	102,6	1,6	-186.1 to 189.3

compound	Difference	t	P value	Summary
Gallic	63,5	1,205	P > 0.05	ns
Catechin	10190	193,3	P<0.001	***
Chlorogenic	-2986	56,65	P<0.001	***
3-OH	134	2,542	P > 0.05	ns
Rutin	-3624	68,75	P<0.001	***
Sinapinic	0	0	P > 0.05	ns
Quercetin	-89,6	1,7	P > 0.05	ns
Carvacrol	1,6	0,03035	P > 0.05	ns

verum vs. pseudoaristatum

compound	verum	pseudoaristatum	Difference	95% CI of diff.
Gallic	0	108,5	108,5	-79.23 to 296.2
Catechin	0	202,6	202,6	14.87 to 390.3
Chlorogenic	2986	1640	-1347	-1534 to -1159
3-OH	853	87,4	-765,6	-953.3 to -577.9
Rutin	3624	282,8	-3341	-3529 to -3153
Sinapinic	0	55,7	55,7	-132.0 to 243.4
Quercetin	89,6	67,9	-21,7	-209.4 to 166.0
Carvacrol	101	91,8	-9,2	-196.9 to 178.5

compound	Difference	t	P value	Summary
Gallic	108,5	2,058	P > 0.05	ns
Catechin	202,6	3,843	P<0.01	**
Chlorogenic	-1347	25,54	P<0.001	***
3-OH	-765,6	14,52	P<0.001	***
Rutin	-3341	63,38	P<0.001	***
Sinapinic	55,7	1,057	P > 0.05	ns
Quercetin	-21,7	0,4117	P > 0.05	ns
Carvacrol	-9,2	0,1745	P > 0.05	ns

verum vs. purpureum

compound	verum	purpureum	Difference	95% CI of diff.
Gallic	0	23,2	23,2	-164.5 to 210.9
Catechin	0	0	0	-187.7 to 187.7
Chlorogenic	2986	5572	2586	2398 to 2774
3-OH	853	374	-479	-666.7 to -291.3
Rutin	3624	137,3	-3487	-3674 to -3299
Sinapinic	0	203	203	15.27 to 390.7
Quercetin	89,6	0	-89,6	-277.3 to 98.13
Carvacrol	101	161,8	60,8	-126.9 to 248.5

compound	Difference	t	P value	Summary
Gallic	23,2	0,4401	P > 0.05	ns
Catechin	0	0	P > 0.05	ns
Chlorogenic	2586	49,06	P<0.001	***
3-OH	-479	9,087	P<0.001	***
Rutin	-3487	66,14	P<0.001	***
Sinapinic	203	3,851	P<0.01	**
Quercetin	-89,6	1,7	P > 0.05	ns
Carvacrol	60,8	1,153	P > 0.05	ns

album vs. rivale

compound	album	rivale	Difference	95% CI of diff.
Gallic	0	63,5	63,5	-124.2 to 251.2

Catechin	380,1	10190	9812	9624 to 10000
Chlorogenic	8310	0	-8310	-8498 to -8122
3-OH	0	987	987	799.3 to 1175
Rutin	275	0	-275	-462.7 to -87.27
Sinapinic	0	0	0	-187.7 to 187.7
Quercetin	84,1	0	-84,1	-271.8 to 103.6
Carvacrol	84,07	102,6	18,53	-169.2 to 206.3

compound	Difference	t	P value	Summary
Gallic	63,5	1,205	P > 0.05	ns
Catechin	9812	186,1	P<0.001	***
Chlorogenic	-8310	157,6	P<0.001	***
3-OH	987	18,72	P<0.001	***
Rutin	-275	5,217	P<0.001	***
Sinapinic	0	0	P > 0.05	ns
Quercetin	-84,1	1,595	P > 0.05	ns
Carvacrol	18,53	0,3515	P > 0.05	ns

album vs. pseudoaristatum

compound	album	pseudoaristatum	Difference	95% CI of diff.
Gallic	0	108,5	108,5	-79.23 to 296.2
Catechin	380,1	202,6	-177,5	-365.2 to 10.23
Chlorogenic	8310	1640	-6671	-6858 to -6483
3-OH	0	87,4	87,4	-100.3 to 275.1
Rutin	275	282,8	7,8	-179.9 to 195.5
Sinapinic	0	55,7	55,7	-132.0 to 243.4
Quercetin	84,1	67,9	-16,2	-203.9 to 171.5
Carvacrol	84,07	91,8	7,73	-180.0 to 195.5

compound	Difference	t	P value	Summary
Gallic	108,5	2,058	P > 0.05	ns
Catechin	-177,5	3,367	P<0.01	**
Chlorogenic	-6671	126,5	P<0.001	***
3-OH	87,4	1,658	P > 0.05	ns
Rutin	7,8	0,148	P > 0.05	ns
Sinapinic	55,7	1,057	P > 0.05	ns
Quercetin	-16,2	0,3073	P > 0.05	ns
Carvacrol	7,73	0,1466	P > 0.05	ns

album vs. purpureum

compound	album	purpureum	Difference	95% CI of diff.
Gallic	0	23,2	23,2	-164.5 to 210.9
Catechin	380,1	0	-380,1	-567.8 to -192.4
Chlorogenic	8310	5572	-2738	-2926 to -2550
3-OH	0	374	374	186.3 to 561.7
Rutin	275	137,3	-137,7	-325.4 to 50.03

Sinapinic	0	203	203	15.27 to 390.7
Quercetin	84,1	0	-84,1	-271.8 to 103.6
Carvacrol	84,07	161,8	77,73	-110.0 to 265.5

compound	Difference	t	P value	Summary
Gallic	23,2	0,4401	P > 0.05	ns
Catechin	-380,1	7,211	P<0.001	***
Chlorogenic	-2738	51,94	P<0.001	***
3-OH	374	7,095	P<0.001	***
Rutin	-137,7	2,612	P > 0.05	ns
Sinapinic	203	3,851	P<0.01	**
Quercetin	-84,1	1,595	P > 0.05	ns
Carvacrol	77,73	1,475	P > 0.05	ns

rivale vs. pseudoaristatum

compound	rivale	pseudoaristatum	Difference	95% CI of diff.
Gallic	63,5	108,5	45	-142.7 to 232.7
Catechin	10190	202,6	-9989	-10180 to -9802
Chlorogenic	0	1640	1640	1452 to 1827
3-OH	987	87,4	-899,6	-1087 to -711.9
Rutin	0	282,8	282,8	95.07 to 470.5
Sinapinic	0	55,7	55,7	-132.0 to 243.4
Quercetin	0	67,9	67,9	-119.8 to 255.6
Carvacrol	102,6	91,8	-10,8	-198.5 to 176.9

compound	Difference	t	P value	Summary
Gallic	45	0,8537	P > 0.05	ns
Catechin	-9989	189,5	P<0.001	***
Chlorogenic	1640	31,1	P<0.001	***
3-OH	-899,6	17,07	P<0.001	***
Rutin	282,8	5,365	P<0.001	***
Sinapinic	55,7	1,057	P > 0.05	ns
Quercetin	67,9	1,288	P > 0.05	ns
Carvacrol	-10,8	0,2049	P > 0.05	ns

rivale vs. purpureum

compound	rivale	purpureum	Difference	95% CI of diff.
Gallic	63,5	23,2	-40,3	-228.0 to 147.4
Catechin	10190	0	-10190	-10380 to - 10000
Chlorogenic	0	5572	5572	5384 to 5760
3-OH	987	374	-613	-800.7 to -425.3
Rutin	0	137,3	137,3	-50.43 to 325.0
Sinapinic	0	203	203	15.27 to 390.7
Quercetin	0	0	0	-187.7 to 187.7
Carvacrol	102,6	161,8	59,2	-128.5 to 246.9

compound	Difference	t	P value	Summary
Gallic	-40,3	0,7645	P > 0.05	ns
Catechin	-10190	193,3	P<0.001	***
Chlorogenic	5572	105,7	P<0.001	***
3-OH	-613	11,63	P<0.001	***
Rutin	137,3	2,605	P > 0.05	ns
Sinapinic	203	3,851	P<0.01	**
Quercetin	0	0	P > 0.05	ns
Carvacrol	59,2	1,123	P > 0.05	ns

pseudoaristatum vs. purpureum

compound	pseudoaristatum	purpureum	Difference	95% CI of diff.
Gallic	108,5	23,2	-85,3	-273.0 to 102.4
Catechin	202,6	0	-202,6	-390.3 to -14.87
Chlorogenic	1640	5572	3933	3745 to 4120
3-OH	87,4	374	286,6	98.87 to 474.3
Rutin	282,8	137,3	-145,5	-333.2 to 42.23
Sinapinic	55,7	203	147,3	-40.43 to 335.0
Quercetin	67,9	0	-67,9	-255.6 to 119.8
Carvacrol	91,8	161,8	70	-117.7 to 257.7

compound	Difference	t	P value	Summary
Gallic	-85,3	1,618	P > 0.05	ns
Catechin	-202,6	3,843	P<0.01	**
Chlorogenic	3933	74,6	P<0.001	***
3-OH	286,6	5,437	P<0.001	***
Rutin	-145,5	2,76	P > 0.05	ns
Sinapinic	147,3	2,794	P > 0.05	ns
Quercetin	-67,9	1,288	P > 0.05	ns
Carvacrol	70	1,328	P > 0.05	ns

Section S2

Chromatograms of dry extracts of *Galium* species obtained using DLLME (@ 278 nm as example of wavelength in which all compounds show absorbance).

Galium verum

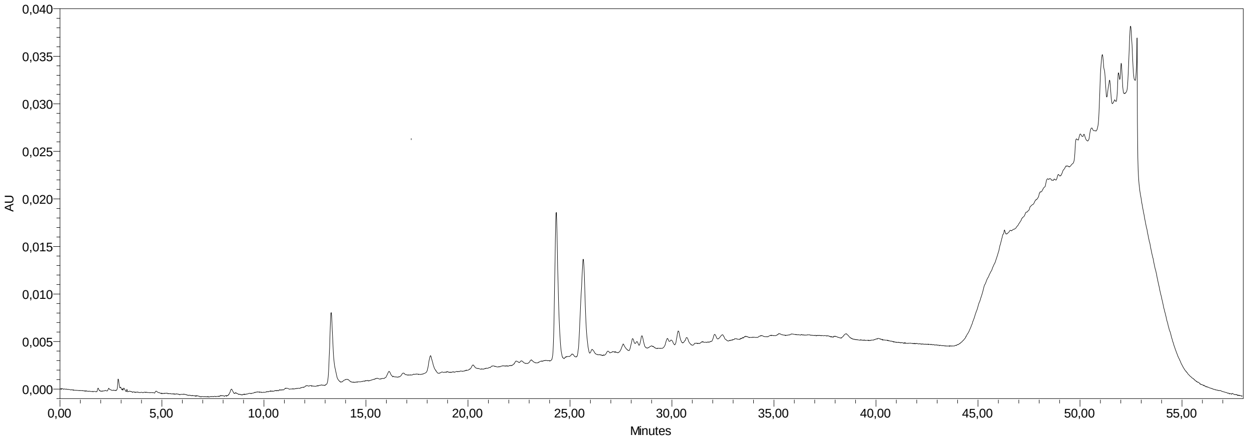

Galium erectum

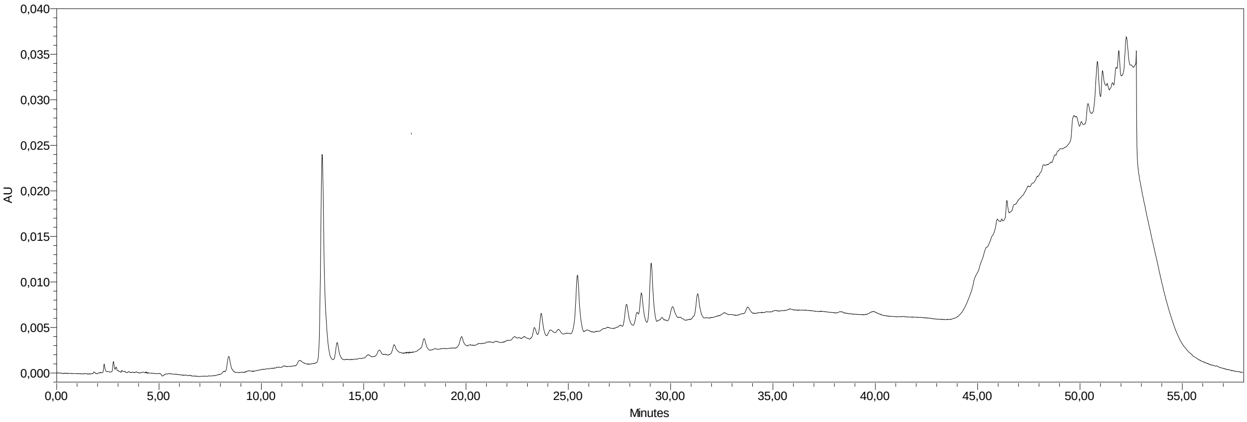

Galium rivale

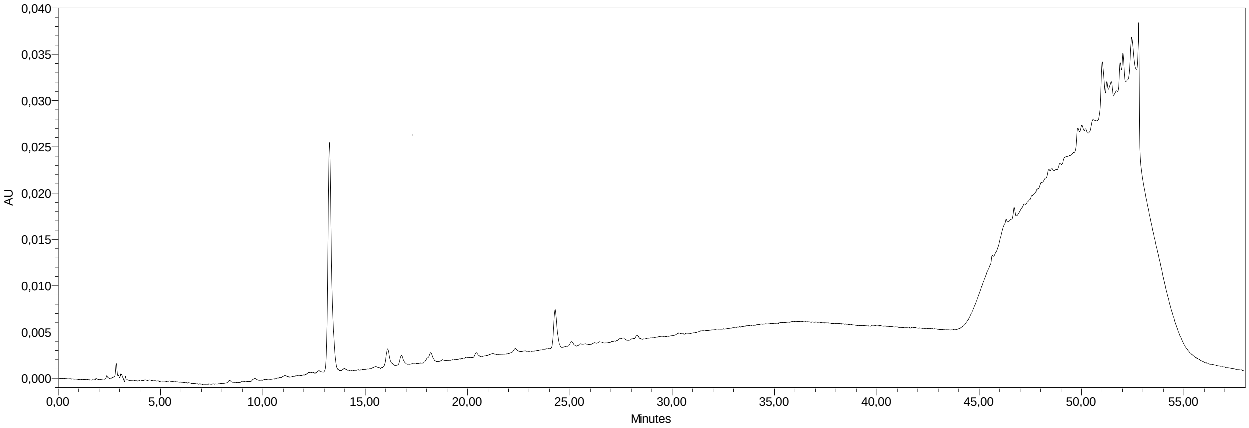

Galium pseudoaristatum

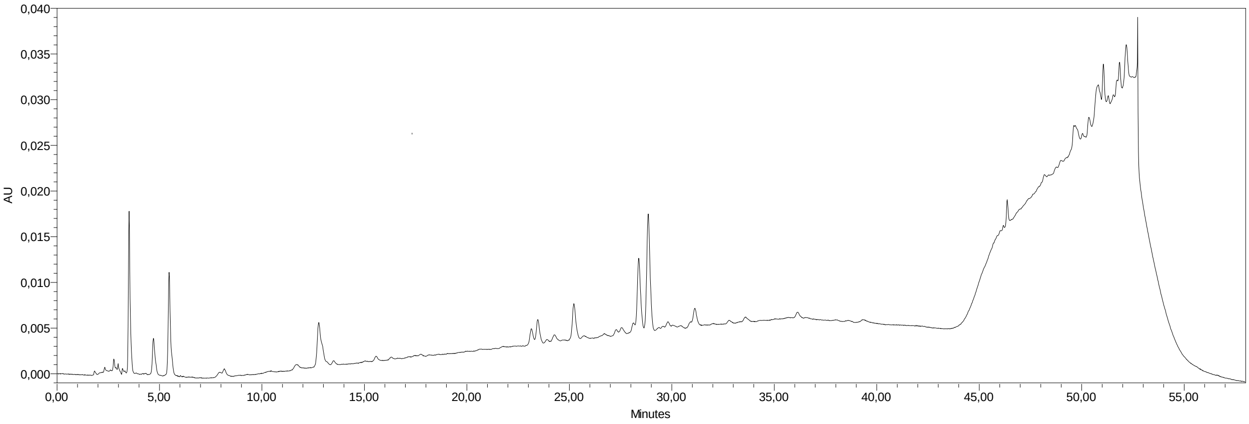

Galium purpureum

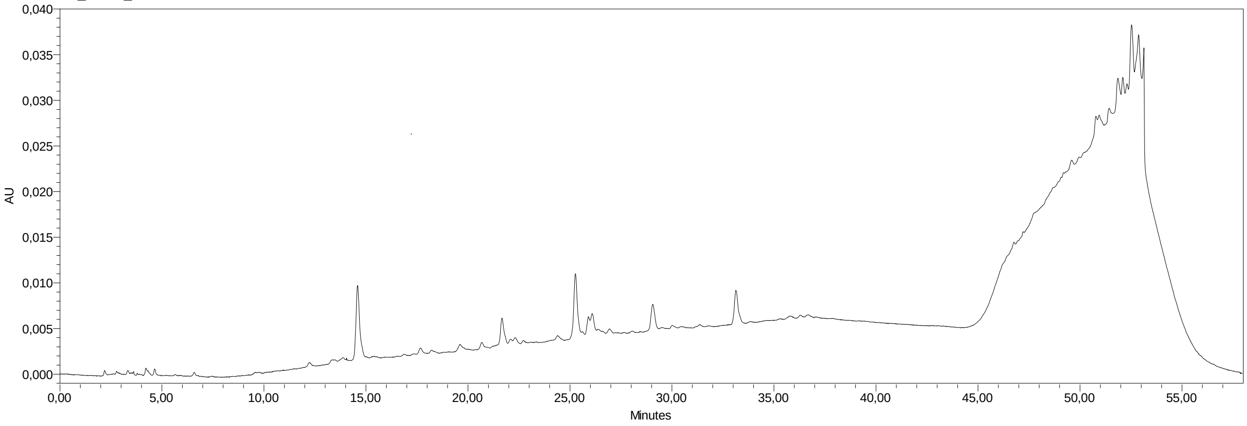

Section S3

Chromatogram of phenolics standards (@ 278 nm as example of wavelength in which all compounds show absorbance) for the 22 analytes, with a table reporting the retention times and the maximum wavelengths used for the quantitative analyses. A table with gradient elution program used for the analyses was also reported.

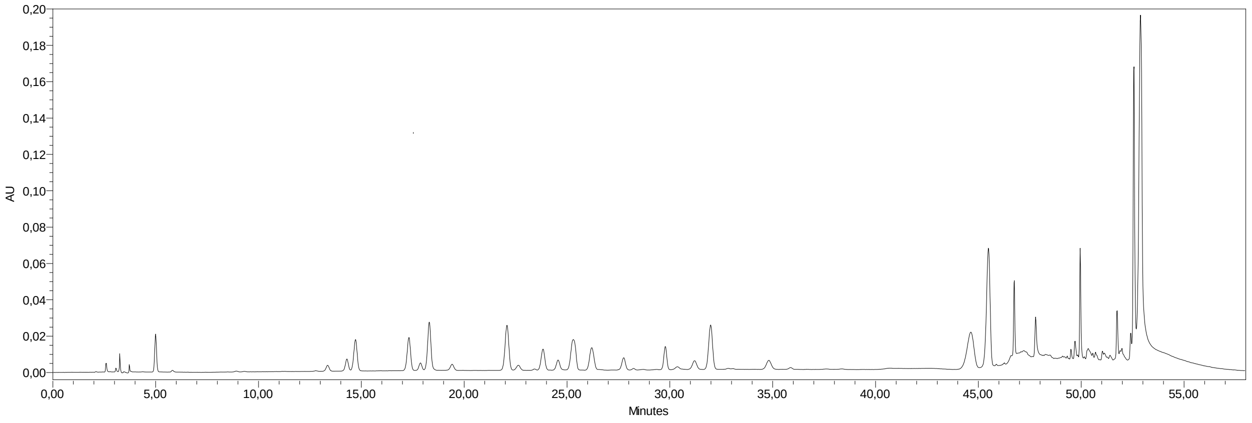

Gradient elution:

TIME (min)	FLOW (mL min^{-1})	%A	%B
0	1	93	7
0.1		93	7
30		72	28
38		75	25
45		2	98
47		2	98
48		93	7
58		93	7

Compound	Wavelength, nm	Retention time, min
Gallic acid	271	4.99
Catechin	278	13.36
Chlorogenic acid	324	14.29
p-OH benzoic acid	256	14.71
Vanillic acid	260	17.31
Epicatechin	278	17.87
Syringic acid	274	18.30
3-OH benzoic acid	295	19.41
3-OH-4-MeO benzaldehyde	275	22.08
p-coumaric acid	309	22.65
Rutin	256	25.38
Sinapinic acid	324	26.18
t-ferulic acid	315	27.75
Naringin	285	29.78
2,3-diMeO benzoic acid	299	30.36
Benzoic acid	275	31.20
o-coumaric acid	276	34.81
Quercetin	367	40.57
Harpagoside	280	45.49
t-cinnamic acid	276	45.87
Naringenin	290	46.74
Carvacrol	275	49.95
